# RelB acts as a molecular switch driving chronic inflammation in glioblastoma multiforme

**DOI:** 10.1038/s41389-019-0146-y

**Published:** 2019-05-29

**Authors:** Michael R. Waters, Angela S. Gupta, Karli Mockenhaupt, LaShardai N. Brown, Debolina D. Biswas, Tomasz Kordula

**Affiliations:** Department of Biochemistry and Molecular Biology, Virginia Commonwealth, University School of Medicine and the Massey Cancer Center, Richmond, VI 23298 USA

**Keywords:** CNS cancer, Inflammation

## Abstract

Glioblastoma multiforme (GBM) is a primary brain tumor characterized by extensive necrosis and immunosuppressive inflammation. The mechanisms by which this inflammation develops and persists in GBM remain elusive. We identified two cytokines interleukin-1β (IL-1) and oncostatin M (OSM) that strongly negatively correlate with patient survival. We found that these cytokines activate RelB/p50 complexes by a canonical NF-κB pathway, which surprisingly drives expression of proinflammatory cytokines in GBM cells, but leads to their inhibition in non-transformed astrocytes. We discovered that one allele of the gene encoding deacetylase Sirtuin 1 (SIRT1), needed for repression of cytokine genes, is deleted in 80% of GBM tumors. Furthermore, RelB specifically interacts with a transcription factor Yin Yang 1 (YY1) in GBM cells and activates GBM-specific gene expression programs. As a result, GBM cells continuously secrete proinflammatory cytokines and factors attracting/activating glioma-associated microglia/macrophages and thus, promote a feedforward inflammatory loop.

## Introduction

Robust angiogenesis, radioresistance, and invasion make glioblastoma multiforme (GBM) one of the most lethal cancers with patient survival rates that have not improved in decades^1,2^. Although GBM tumors exhibit mutations in known tumor suppressors and oncogenes, they are extensively heterogeneous^3^. Unsupervised clustering analysis of GBM tumors identified four distinct gene expression subtypes, which are now recognized as proneural, neural, classical, and mesenchymal GBMs^4,5^. These transcriptional subtypes are associated with frequent specific somatic alternations, such as PDGFRA amplifications, and IDH1 and TP53 mutations in the proneural subtype, EGFR alterations in the classical subtype, and NF1 abnormalities in the mesenchymal GBM^5,6^. While inflammation also develops in all GBM subtypes, extensive necrosis and profound immunosuppressive inflammation characterizes the most common and deadly mesenchymal subtype of GBM, which is more resistant to standard therapies and has the worst prognosis^7,8^. The unique immunosuppressive microenvironment is the major obstacle for immunotherapy, which so far has not been successful in GBM^9–13^. GBM tumors are extensively infiltrated by immune cells, including glioma-associated microglia/macrophages (GAMs), myeloid-derived suppressor cells, T cells, and granulocytes^14^. Additionally, expression of cytokines, chemokines, and other inflammatory mediators produced by both GBM cells and GBM microenvironment is most robust in the mesenchymal GBM tumors^15^. The proinflammatory signaling induced by these mediators enhances the proliferation, invasiveness, resistance to apoptosis, maintenance of stem cell-like properties, drug resistance of GBM cells, and angiogenesis of GBM tumors, driving tumor progression^16,17^. Despite significant effects on tumor progression, mechanisms by which this immunosuppressive inflammation develops in GBM and persists regardless of multiple anti-inflammatory feedback mechanisms remain elusive.

Inflammation is associated with activation of the NF-κB family of transcription factors (p65, cRel, p105/p50, p100/p52, and RelB) that regulate a wide-range of processes, including cell survival and both immune and inflammatory responses^18^. Classically, NF-κB proteins are activated either via a canonical or a non-canonical pathway leading to the activation of p65/p50 or RelB/p52 heterodimers, respectively. Although the p65/p50 complexes are activated by many proinflammatory stimuli, the RelB/p52 complexes are activated by a selected group of ligands, and control development of lymphoid organs^19^. It has been proposed that non-canonical pathway-activated RelB/p52 complexes can promote GBM progression^20–23^. However, although aberrant p65/p50 and RelB/p52 signaling have been linked to oncogenesis and progression^24^, targeting these pathways has not been beneficial^24^, and inhibitors, such as sulfasalazine have failed clinical trials in GBM^25^. Interestingly, however, RelB can also form RelB/p50/IκBα complexes, activated by canonical stimuli, in cells expressing high levels of RelB^26^. RelB also limits inflammation in innate immune cells and astrocytes by several mechanisms^27–29^. Paradoxically, RelB is also a marker of the highly inflammatory mesenchymal GBM subtype^5^. Given RelB’s key role in establishing a negative inflammatory feedback in astrocytes^29^, we asked whether its activation by the canonical NF-κB pathway regulates inflammation associated with mesenchymal GBM.

## Results

### IL-1 and OSM specifically predict short GBM patient survival

**Fig. 1:**
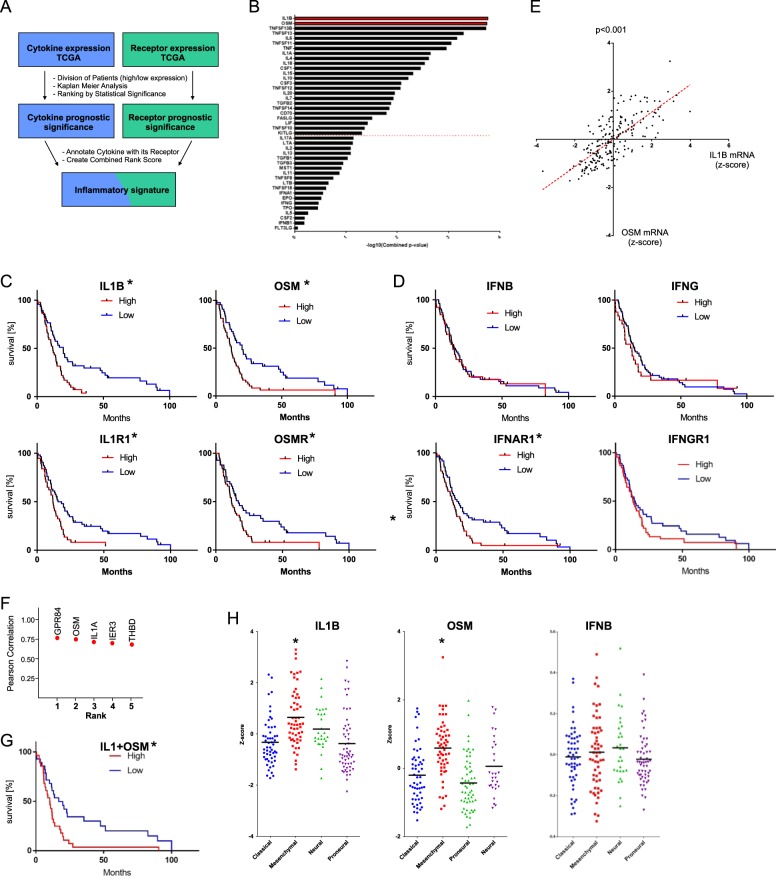
IL-1 and OSM specifically predict short GBM patient survival. **a** Workflow of CytoAnalysis to establish inflammatory signature of GBM patients (TCGA, *n* = 208). For each cytokine and cytokine receptor gene, the mean *Z*-score normalized expression values were used to group patients into high and low expressors. Kaplan–Meier analysis was performed yielding a *p*-value indicating the prognostic relationship between each gene and survival. These *p*-values were used to rank cytokines and cytokine receptors as indicators of poor prognosis. A combined prognostic score was then generated for each cytokine and its receptor. **b** Inflammatory signature (combined rank score) of GBM tumors determined by CytoAnalysis. **c**, **d** Kaplan–Meier analysis for individual cytokines and their receptors. Patients were annotated as high and low expressors using the mean *Z*-score expression level as a cutoff. Statistical significance was assessed using the Cox-proportional hazards model, *p < 0.05. **e** Pearson correlation of IL-1β and OSM mRNA expression scores for GBM patients (TCGA, *n* = 208). Normalized *Z*-score expression values were downloaded and Pearson correlation was conducted using cBioPortal. Both *p*-value and regression analysis was performed using the ‘lm’ function in the core R statistical package. **f** Genome-wide gene rank of Pearson correlation with *IL1B*. Gene expression correlations were performed using cBioPortal. **g** Kaplan–Meier survival analysis of patients expressing either high or low levels of both IL-1β and OSM, analyzed as in **c**. *p < 0.05. **h** Patient subtype was downloaded using clinical expression information contained within TCGA (*n* = 206). *p < 0.05; One-way ANOVA

**Fig. 2:**
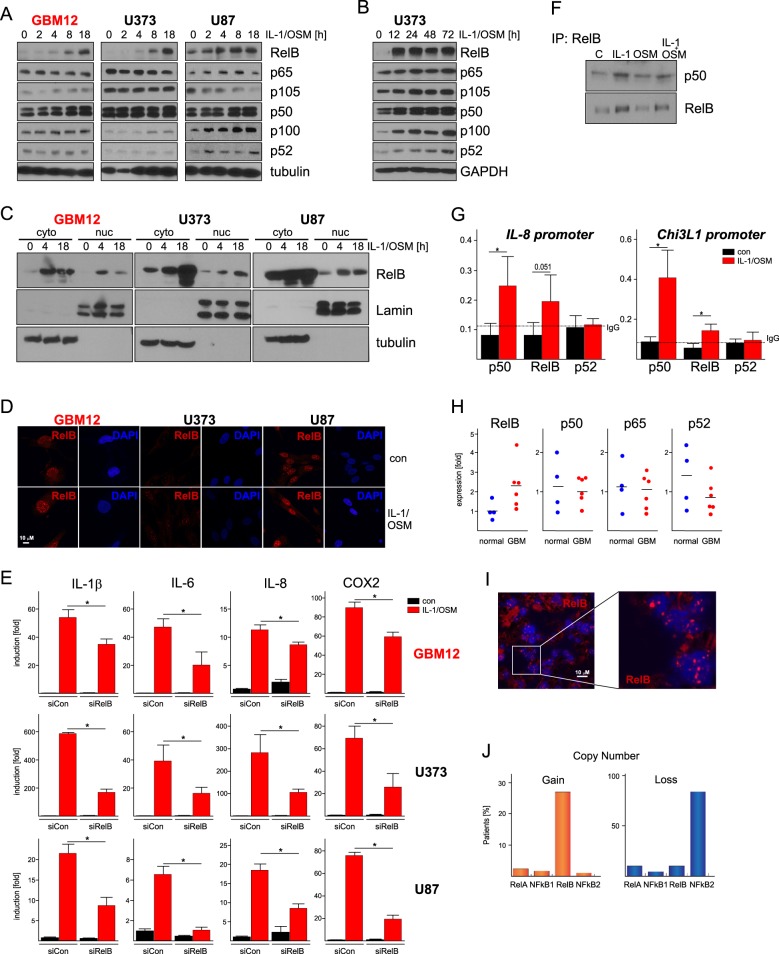
IL-1/OSM activate RelB/p50 in GBM. **a** Primary GBM12 cells and established U373 and U87 cell lines were stimulated with IL-1/OSM as indicated. Expression of RelB, p65, p105, p50, p100, and p52 was analyzed by western blotting. Tubulin was used as a loading control. **b** U373 cells were treated with IL-1/OSM as indicated, expression was assessed by western blotting. **c** Nuclear and cytosolic fractions were prepared from control or IL-1/OSM-stimulated cells (at indicated time points), and RelB expression was analyzed by western blotting. Lamin A/C and tubulin were used to examine purity of the fractions. **d** Cells were stimulated for 18 h, and RelB visualized by immunofluorescence. DAPI was used to stain nuclei. **e** Cells transfected with the indicated siRNAs were stimulated 48 h later with IL-1/OSM for 18 h. Expression was analyzed by qPCR (three experiments, error bars represent s.d., *p < 0.05 (two-way ANOVA, Sidak’s test). **f** U373 were stimulated for 8 h, RelB was immunoprecipitated, and p50 and RelB were detected by western blotting. **g** Binding of RelB, p50, and p52 at the indicated cytokine promoters was analyzed by ChIP. U373 cells were stimulated with IL-1/OSM for 8 h. Normalized binding is shown. IgG was used as a control for IP (dotted line). *n* = 3–6, error bars represent s.d., *p < 0.05 (*T*-test, Sidak’s test). **h** Expression was analyzed by qPCR in human GBM tumors (*n* = 6) and normal brains (*n* = 4). **i** RelB was visualized in tumor sample of GBM patient by immunofluorescence. DAPI was used to counterstain nuclei. **j** Genomic copy number analysis of NF-κB family members, GISTIC data downloaded from TCGA via cBioPortal (*n* = 206)

Although proinflammatory cytokines are secreted transiently during acute inflammation, chronic immunosuppressive inflammatory state develops in GBM and promotes tumor progression^16,17^. To identify cytokines which specifically support GBM aggressiveness, we used an unbiased approach and correlated cytokine and cytokine receptor expression with clinical outcome data from The Cancer Genome Atlas (TCGA) (Fig. 1a, b, Supplementary Table 1). We identified two cytokines interleukin-1β (IL-1) and oncostatin M (OSM) (and their receptors) (Fig. 1c) that most strongly negatively correlated with patient survival, in contrast to many others that did not, such as interferon β (IFNβ) and IFNγ (Fig. 1d). Interestingly, expression of IL-1 strongly correlated with expression of OSM (Pearson correlation = 0.769) (Fig. 1e). Remarkably, ranking of every expressed gene in GBM, according to their Pearson correlation, indicated that the *OSM* gene is the second most correlated with the *IL1B* gene in the entire genome (Fig. 1f), and patients expressing high levels of both cytokines have very poor prognosis (Fig. 1g). Additionally, IL-1 and OSM are expressed at high levels in mesenchymal GBM (Fig. 2h). Since IL-1 is expressed by GBM tumors^30^, GAMs, microglia, and reactive astrocytes^31^, while OSM is produced only by macrophages and microglia^32^, we hypothesized that chronic elevation of IL-1 and OSM levels initiates programs driving GBM progression.

### RelB/p50 canonical signaling in GBM

Since canonical NF-κB stimuli, such as IL-1, can activate RelB/p50 complexes^26,29,33^, we tested whether these complexes are activated by IL-1/OSM in GBM cells. Similarly to what we previously found in astrocytes^29,33^, basal expression of RelB is low in GBM cells. However, IL-1/OSM induced expression of RelB and p50 in both primary GBM cells and established GBM cell lines (Fig. 2a, b). Similarly to p65 (Supplemewntary Fig. 1a), RelB translocated to the nucleus of the GBM cells in response to IL-1/OSM (Fig. 2c). RelB formed distinctive puncta in the nuclei of GBM cells (Fig. 2d, Supplementary Fig. 1b), and its expression was p65-dependent (Suppementary Fig. 1c). Strikingly, while knockdown of RelB (Supplementary Fig. 2a) enhanced expression of proinflammatory cytokines and COX-2 in primary astrocytes (Supplementary Fig. 2b), it had an opposite effect in primary GBM cells and established GBM cell lines (Fig. 2e). Importantly, IL-1 (alone or together with OSM) induced the formation of RelB/p50 complexes (Fig. 2f). Both RelB and p50, but not p52, bound the target promoters (Fig. 2g). RelB expression was higher in GBM samples than normal brains (Fig. 2h), and RelB was also almost entirely localized in the nuclei in GBM patient samples (Fig. 2i). While previous reports showed that RelB/p52 complexes promote GBM progression^20–23^, we strikingly found that over 80% of GBMs lost one allele of the NFKB2 (p100/p52), but almost 30% of GBMs have RelB allele gains (Fig. 2j). In summary, in response to IL-1/OSM, RelB/p50-canonical signaling is activated in GBM cells in vitro, and RelB-canonical signaling is likely more prevalent in GBM in vivo.

### Opposing effects of RelB in GBM cells versus astrocytes

**Fig. 3:**
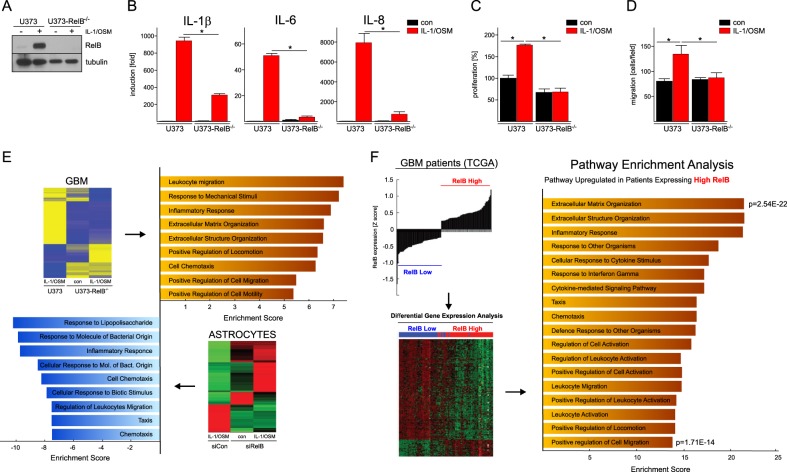
Opposing effects of RelB in GBM cells versus astrocytes. Parental U373 or U373-RelB^−/−^ cells were treated with IL-1/OSM for 18 h and **a** expression assessed by western blotting; **b** expression analyzed by qPCR. *p < 0.05 (three experiments, error bars represent s.d., two-way ANOVA, Sidak’s test); **c** proliferation (*p < 0.05, *n* = 3, error bars represent s.d., two-way ANOVA, Sidak’s test); **d** migration (*p < 0.05, *n* = 3, error bars represent s.d., two-way ANOVA, Sidak’s test). **e** (Top panel) RNA-seq analysis of U373 or U373-RelB^−/−^ cells untreated/treated with IL-1/OSM (18 h) was performed. Pathway enrichment analysis is shown. (Bottom panel) Primary human astrocytes transfected with the indicated siRNAs were stimulated 48 h later with IL-1/OSM for 18 h. Microarray analysis was performed. Pathway enrichment analysis is shown. **f** Patients (*n* = 206) were classified as high and low RelB expressers, and differential gene expression testing was conducted. Genes upregulated in the patients expressing high RelB levels were used for pathway enrichment analysis

To gain insight into RelB-driven gene expression programs in GBM, we generated U373-RelB-deficient cells using CRISPR/CAS9 (Fig. 3a). Similarly to RelB knock-down (Fig. 2e), knock-out of RelB severely diminished IL-1/OSM-induced cytokine expression (Fig. 3b) and also diminished cytokine-induced proliferation (Fig. 3c) and migration (Fig. 3d). In order to define the global role of RelB in GBM cells, we performed RNA-seq analysis of parental and RelB-deficient cells, and conducted differential expression testing and pathway enrichment analysis. RNA-seq analysis indicated that in response to cytokines, RelB overwhelmingly activates genes in GBM, and the enriched pathways are involved in proinflammatory responses (*p* = 1.37 × 10^−7^), perpetuating inflammation, and inflammatory cell chemotaxis (Fig. 3e). Conversely, using the RelB knock-down approach, we found that RelB inhibits proinflammatory response (*p* = 1.92 × 10^−10^) in primary astrocytes (Fig. 3e). Thus, this genome-wide expression analysis suggests that RelB functions mostly as a transcriptional repressor in astrocytes, but as a transcriptional activator in GBM cells in vitro. To test whether RelB functions as a transcriptional activator in GBM in vivo, we performed differential expression testing and pathway enrichment analysis of patients expressing either high or low levels of RelB. Interestingly, patients expressing high RelB levels show enrichment of the same pathways (Fig. 3f), which were activated by RelB in GBM cells in vitro (Fig. 3e).

### RelB coordinates recruitment and activation of myeloid cells in GBM

**Fig. 4:**
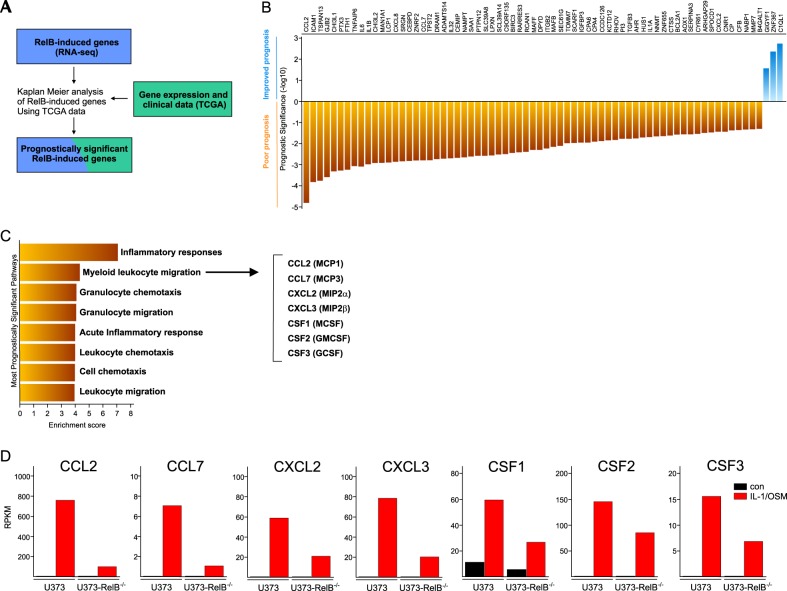
RelB regulates recruitment and activation of GAMs. **a** Workflow of RelB effect on clinical outcome of GBM patients. Independent effect of RelB-controlled genes on patient prognosis was evaluated using gene expression data and clinical outcome data downloaded from TCGA. **b** Prognostic significance of RelB-controlled genes (workflow **a**), which have a statistically significant impact on GBM patient prognosis. **c** Pathway enrichment analysis of RelB-controlled genes significantly impacting prognosis. **d** Expression of prognostically significant RelB-controlled genes annotated to function as macrophage chemoattractants/activators. FPKM fragments per kilobase of transcript per million mapped reads (RNA-seq data)

To determine which of the RelB-dependent genes identified by RNA-seq have the greatest impact on patient survival, we wrote an in house R-script to perform “iterative Kaplan–Meier analysis” (Fig. 4a). Importantly, the vast majority of RelB-dependent genes, statistically important for patient prognosis, are markers of poor prognosis (Fig. 4b). Pathway enrichment analysis for these genes showed that the inflammatory genes including those chemotactic for myeloid cells are the most overrepresented in patients expressing high levels of RelB (Fig. 4c). These data suggested that a major effect of RelB programs in GBM may be recruitment of myeloid cells, which leads to more aggressive tumors^34^. Although RelB did not induce classical drivers of the M1 (IFN*y* and TNF*α*) or the M2 phenotype (IL-4, IL-13), prognostically important genes specifically induced by RelB are *CSF1, CSF2, CSF3, CCL2, CCL7, CXCL2*, and *CXCL3* (Fig. 4d). Since proteins encoded by these genes are known to activate and attract myeloid cells to the sites of inflammation, RelB may be controlling recruitment and activation of GAMs.

### Loss of SIRT1 is associated with persistent inflammation in GBM

**Fig. 5:**
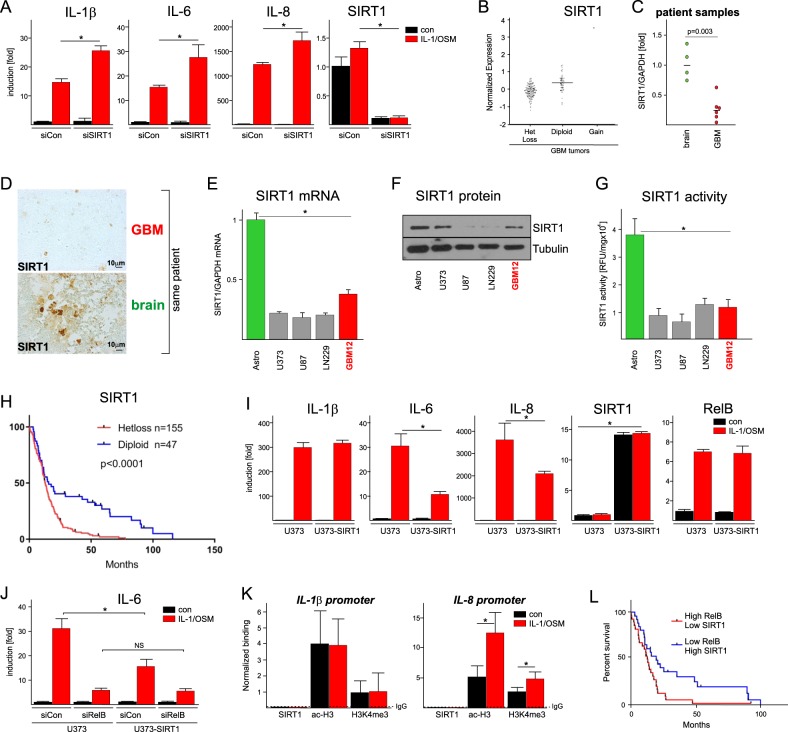
Loss of SIRT1 expression/activity in GBM diminishes patient survival. **a** Primary human astrocytes transfected with the indicated siRNAs were stimulated 48 h later with IL-1/OSM for 18 h, and expression was analyzed by qPCR. *n* = 3, error bars represent s.d., *p < 0.05 (two-way ANOVA, Sidak’s test). **b** Plot of SIRT1 zygosity and expression in GBM tumors. *Z*-score normalized SIRT1 expression values were correlated with patients SIRT1 zygosity established via GISTIC analysis. **c** Expression of SIRT1 was analyzed by qPCR in brain tissue (*n* = 4) and GBM tumors (*n* = 6). *p*-value is indicated (one-way ANOVA, *t*-test). **d** SIRT1 protein was visualized by immunohistochemistry in GBM tumor and normal brain tissue (same patient). **e** Expression of SIRT1 mRNA was examined by qPCR in primary human astrocytes, GBM cell lines, and primary GBM12 cells. *n* = 3, error bars represent s.d., *p < 0.05 (one-way ANOVA, Sidak’s test). **f** Expression of SIRT1 protein was examined by WB in primary human astrocytes, GBM cell lines, and primary GBM12 cells. Representative image is shown. **g** SIRT1 enzymatic activity was analyzed using fluorescence substrate. *n* = 3, error bars represent s.d., *p < 0.05 (one-way ANOVA, Sidak’s test). **h** Kaplan–Meier analysis of patients’ survival (*n* = 206). Patients with diploid SIRT1 allele, and heterozygous loss (Hetloss) were identified as in **b**. **i** Expression of cytokines, RelB, and SIRT1 mRNA was examined by qPCR in parental and SIRT1 overexpressing U373 cells. *n* = 3, error bars represent s.d., *p < 0.05 (two-way ANOVA, Sidak’s test). **j** Cells transfected with the indicated siRNAs were stimulated 48 h later with IL-1/OSM for 18 h. Expression was analyzed by qPCR (*n* = 3, error bars represent s.d., *p < 0.05 (two-way ANOVA, Sidak’s test). **k** Acetylation of histone H3 (ac-H3), tri-methylation of histone H3 on lysine K4 (H3K4me^3^), and presence of SIRT1 at the indicated cytokine promoters was analyzed by ChIP. U373 cells were stimulated with IL-1/OSM for 8 h. Normalized binding is shown. IgG was used as a control for IP (dotted line). *n* = 3, error bars represent s.d., *p < 0.05 (two-way ANOVA, Sidak’s test). **l** Kaplan–Meier analysis of patients’ survival (*n* = 206). Patients expressing high/low RelB and SIRT one were defined as in Fig. 3f

NAD^+^-dependent histone deacetylase SIRT1 has been implicated in RelB-mediated epigenetic silencing that regulates LPS tolerance in macrophages^35,36^. SIRT1 also regulates adaptive responses of astrocytes by suppressing IL-1-induced activation of cytokine genes^29^. Although SIRT1 suppressed IL-1/OSM-induced cytokine expression in astrocytes (Fig. 5a), it surprisingly had no effect in GBM cells (Supplementary Fig. 3a). Mining of TCGA database showed that one allele of the *SIRT1* gene is deleted in ~80% of GBM tumors (Fig. 5b). We also found lower expression of SIRT1 mRNA in GBMs than normal (matching) brain (Fig. 5c), and confirmed these patient’s samples on the protein level by IHC (Fig. 5d). These findings were further confirmed in vitro since SIRT1 mRNA levels (Fig. 5e), SIRT1 protein (Fig. 5f), and SIRT1 activity (Fig. 5g) were decreased in GBM cell lines and primary GBM cells in comparison to astrocytes. Remarkably, loss of one allele of the *SIRT1* gene leads to poor patient survival (Fig. 5h) (disease-free survival is 4.9 months in comparison to 22 months for diploid patients). We addressed the importance of SIRT1 in RelB-mediated regulation by overexpression of SIRT1 in GBM cells, which significantly diminished expression of IL-6 and IL-8 but had no effect on IL-1 (Fig. 5i). Importantly, the effect of SIRT1 was RelB-dependent, since SIRT1 overexpression was not effective in the absence of RelB (Fig. 5j). Overexpression of SIRT1 also decreased rate of glycolysis in GBM cells (Supplementary Fig. 3b). Although SIRT1 can deacetylate histones, acetylation of histones was not diminished at the cytokine promoters in response to IL1/OSM in GBM cells (Fig. 5k). SIRT1 was also absent at these promoters even though they were active, as indicated by tri-methylation of histone H3 at lysine 4 (Fig. 5k). Significantly, patients expressing RelB at high levels but SIRT1 at low levels have very poor survival prognosis (Fig. 5l). These data suggest that although SIRT1 represses cytokine genes in astrocytes, RelB/SIRT1-dependent repression does not function in GBM cells due to lower expression/activity of SIRT1.

### Yin Yang 1 (YY1) specifically upregulates cytokine expression in GBM cells

**Fig. 6:**
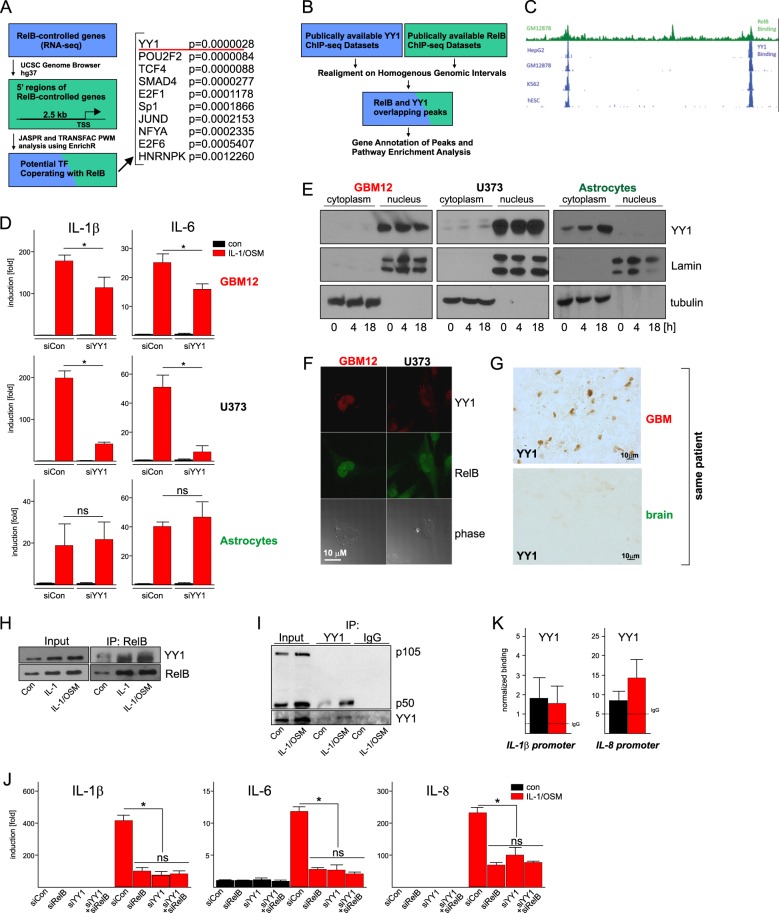
YY1 upregulates cytokine expression in GBM but not astrocytes. **a** Workflow for the identification of putative transcription factors cooperating with RelB (left panel). Rank list of regulatory elements in RelB-controlled genes. *p*-values were directly generated by PWM. **b** Workflow for YY1 and RelB ChIPseq data processing. **c** Representative RelB/YY1 peaks. **d** Cells transfected with the indicated siRNAs were stimulated 48 h later with IL-1/OSM for 18 h. Expression was analyzed by qPCR. *n* = 3, error bars represent s.d., *p < 0.05 (two-way ANOVA, Sidak’s test). **e** Nuclear and cytosolic fractions were prepared from control or IL-1/OSM-stimulated cells (at indicated time points), and YY1 expression was analyzed by western blotting. Lamin A/C and tubulin were used to examine purity of the fractions. **f** Cells were stimulated for 18 h, and YY1 and RelB visualized by immunofluorescence. **g** YY1 protein was visualized by immunohistochemistry in GBM tumor and normal brain tissue (same patient). **h**, **i** U373 cells were stimulated with IL-1 and/or OSM for 18 h. **h** RelB was immunoprecipitated. RelB and YY1 were detected in immunoprecipitates by western blotting. **i** YY1 was immunoprecipitated. p50, p105, and YY1 were detected in immunoprecipitates by western blotting. Expression in the lysates is shown (Input). **j** Cells transfected with the indicated siRNAs were stimulated 48 h later with IL-1/OSM for 18 h. Expression was analyzed by qPCR. *n* = 3, error bars represent s.d., *p < 0.05 (two-way ANOVA, Sidak’s test). **k** YY1 binding at the cytokine promoters was analyzed by ChIP. U373 cells were stimulated with IL-1/OSM for 8 h. Normalized binding is shown. IgG was used as a control for IP (dotted line). *n* = 3

Although lower expression/activity of SIRT1 could explain lack of cytokine gene silencing by RelB in GBM cells, it could not explain “aberrant” RelB-dependent activation of cytokine expression in these cells. We hypothesized that this activation may depend on an additional transcription factor that affects RelB activity in GBM cells but not astrocytes. To identify this factor, we analyzed 2.5 kb-long promoter regions of the RelB-dependent genes for the presence of regulatory elements using the EnrichR algorithm (Fig. 6a)^37^. We identified several regulatory elements, including a motif that binds YY1, a GLI-Krüppel-related zinc-finger transcription factor regulating formation of enhancer-promoter loops, recruitment of corepressors and coactivators, and thus shaping chromatin structure^38^. Indeed, analysis of publicly available ChIP-seq datasets (Fig. 6b) indicated presence of overlapping peaks for both RelB and YY1 at multiple genomic locations, including the *IL1B* gene (Fig. 6c). To test whether YY1 differentially functions in GBM cells versus astrocytes, we depleted YY1 in these cells. We found that although cytokine expression is YY1-independent in astrocytes, it is upregulated by YY1 in GBM cells (Fig. 6d). Significantly, we found that YY1 is localized almost exclusively to cytoplasm of astrocytes, whereas it is almost entirely nuclear in GBM cells (Fig. 6e, f, Supplementary Fig. 4a), which directly correlates with YY1-dependent regulation of cytokine expression. YY1 was also present in the nuclei of GBM cells in tumors, while its localization was mostly cytoplasmic in nearby normal tissue (Fig. 6g). Since YY1 can form complexes with RelB and Oct-2^39^, and Oct-2 elements are the second most enriched motifs in RelB-regulated genes (POU2F2, Fig. 6a), we tested whether YY1 interacts with RelB and p50 in GBM cells. We could co-IP YY1 with RelB (Fig. 6h), and p50, but not p105 (Fig. 6i). YY1 also colocalized with RelB in the nuclei of GBM cells (Fig. 6f). Furthermore, knock-down of YY1 together with RelB (Supplementary Fig. 4b) did not have an additive effect on cytokine expression (Fig. 6j), suggesting that they function together. YY1 was also bound to the cytokine promoters in GBM cells (Fig. 6k). We concluded that nuclear YY1 is likely responsible for specific upregulation of cytokine expression by RelB in GBM cells.

## Discussion

Various components of the non-canonical NF-κB-signaling pathway, including TWEAK, cIAP1/2, NIK, and RelB, have been implicated in tumorigenesis^40^, and these proteins also promote glioma cell invasion^20–23,41^. In contrast to RelB/p52 complexes, the less understood RelB/p50 complexes are activated by the canonical pathway, limit inflammation in innate immune cells, control adaptive responses in astrocytes, are not easily removed from DNA, and provide long-lasting effects^19,29,36^, but they have not been shown to play any role in GBM. Our data indicate that the inflammatory milieu of GBM is rich in cytokines that are known to activate RelB/p50 complexes in normal cells^33^. We further show that RelB/p50 complexes are also activated in GBM cells, suggesting an additional previously unidentified mechanism by which RelB affects GBM biology and patient survival. The importance of the canonical RelB/p50 signaling is further supported by a significant increase in genomic dose of the *RELB* gene (30% of GBM patients) but striking 80% loss of the *NFKB2* (encoding p100/p52). These data strongly support the idea that p52-independent RelB signaling is critical in GBM development or progression.

Our previous^29^ and current data show that cytokine-induced RelB/p50 complexes suppress expression of the cytokine genes in astrocytes. This is in agreement with the silencing role of RelB in macrophages^36^ and microglia^42^. While silencing of the cytokine genes in macrophages and astrocytes depends on RelB and SIRT1^36,43^, proposed repressive mechanisms are different with the role of SIRT1 being elusive in astrocytes. In contrast, we show that RelB supports cytokine gene expression in GBM cells, while SIRT1 has no effect. Although we found astonishingly frequent loss of one allele of the *SIRT1* gene in GBM tumors and diminished expression of SIRT1 in GBM cells, the activity of SIRT1 may be further diminished by high redox status of GBM cells^44^ or by post-translational modifications of SIRT1^45^. These mechanisms are all likely responsible for the diminished activity of SIRT1 in GBM tumors and cells.

Activation of the cytokine genes by RelB in GBM cells is the surprising result indicating that RelB acts as a molecular switch converting RelB-dependent silencing into transcriptional activation fueling immunosuppressive inflammation. We identified YY1 as a unique modifier of RelB-dependent functions in GBM cells. Although YY1 forms complexes with RelB that may directly affect RelB functions, it is also possible that YY1 cooperates with RelB by forming active chromatin loops^38^. However, functions of YY1 are restricted to actively proliferating cells since YY1 is sequestered to the cytoplasm of non-dividing cells by Retinoblastoma protein^46^. Interestingly, YY1 has also been linked to oncogenesis, and its targeting may be beneficial^47^. YY1 has opposing effects to p65/p50 on gene expression^48^, and its expression can be induced by RelB^49^. It remains to be established whether both RelB and YY1 affect tumor generation or progression, but the later seems to be more likely based on our data.

Our unbiased approach identified prognostically important RelB-dependent genes in GBM. In general, RelB supports multiple aspects of inflammation, including cytokine production by GBM cells. However, we speculate that the main outcome of the RelB-controlled program in GBM is the recruitment and activation of GAMs into tumors, which may be critical since increased myeloid cell infiltration is a marker of aggressive disease. GAMs were originally proposed to have an M2 signature^50^, but recently they have been shown to resemble the undifferentiated but active M0 phenotype^51^. Although GBM cells do not secrete classical drivers of the M1 or M2 phenotypes (IFNγ, TNFα, IL-4, and IL-13), RelB in GBM cells supports expression of known activators and chemoattractants of GAMs, including members of the macrophage inflammatory protein (MIP), colony-stimulating factor (CSF), and the macrophage chemotactic protein (MCP) families^50,52^.

In conclusion, we propose that although RelB coordinates anti-inflammatory feedback in astrocytes^29^, this mechanism does not function in GBM cells due to both the limited activity of SIRT1 and the presence of YY1 in the nuclei. As a result, GBM cells continuously secrete cytokines and factors attracting/activating GAMs, and thus promote a feedforward immunosuppressive inflammatory loop. Our studies provide a paradigm shift on the role of RelB in GBM. The RelB/p50 complex may emerge as a new target for future interventions to control GBM, and likely other malignances associated with RelB-dependent chronic inflammation.

## Matherials and methods

### Cell culture and stimulation

Human cortical astrocyte cultures were established using cerebral tissue provided by Advanced Bioscience Resources, and the protocol for obtaining postmortem fetal neural tissue complied with the federal guidelines for fetal research and with the Uniformed Anatomical Gift Act. Astrocytes were cultured as described previously^33,53^. Human glioblastoma U373-MG cells were obtained from American Type Culture Collection, whereas human glioma U87 cells were obtained from Dr. Jaharul Haque (Cleveland Clinic Foundation). Primary GBM12 cells were obtained from Dr. Paul Dent (Virginia Commonwealth University). Cells were cultured in DMEM supplemented with 10% FBS, antibiotics, sodium pyruvate, and non-essential amino acids. Cells were stimulated with 25 ng/ml OSM (R&D Systems) and 10 ng/ml IL-1β (Peprotech).

### Knockdown

Expression was down-regulated using SmartPool siRNAs transfected with Dharmafect 1 (Dharmacon), according to the manufacturer’s instructions. The following sequences were targeted: (RelB; CAUCAGAGCUGCGGAUUUG, GCCCGUCUAUGACAAGAAA, GCACAGAUGAAUUGGAGAU, and GUACCUGCCUCGCGACCAU), (SIRT1; GUACAAACUUCUAGGAAUG, GUAGGCGGCUUGAUGGUAA, GCGAUUGGGUACCGAGAUA, and GGAUAG GUCCAUAUACUUU).

### Quantitative qPCR

RNA was isolated using Trizol (Invitrogen) and 1 μg was reverse-transcribed using the High Capacity cDNA Archive kit (Applied Biosystems). Expression levels were determined using primer-probe sets and TaqMan Universal PCR Master Mix (Applied Biosystems). The cDNAs were diluted 10-fold (target genes) or 100-fold (GAPDH). Gene expression levels were normalized to GAPDH mRNA levels, and presented as a fold induction.

### Western blotting

The cells were lysed in 10 mM Tris (pH 7.4), 150 mM sodium chloride, 1 mM EDTA, 0.5% Nonidet P-40, 1% Triton X-100, 1 mM sodium orthovanadate, 0.2 mM PMSF, and protease inhibitor mixture (Roche Applied Science). Samples were separated using SDS–PAGE and transferred onto nitrocellulose membranes. The anti-β-tubulin (sc-9104), anti-RelB (sc-226), anti-p65 (sc-372), anti-p105/p50 (sc-8414), anti-IκBα (sc-371), and anti-SIRT1 (sc-15404) antibodies (Santa Cruz Biotechnology); anti-Lamin A/C (2032), anti-myc (2276), and anti-p52 (4882) (Cell Signaling); anti-YY1 (A302-779A) (Bethyl Laboratories), and anti-flag (F1804) antibodies (Sigma-Aldrich) were used. Antigen–antibody complexes were visualized by ECL using Immobilon Western blotting kit (Millipore).

### Immunoprecipitation

Two hundred to three hundred micrograms of protein lysates, were pre-cleared with 10 μl of the protein G-Sepharose beads (GE Healthcare) for 1 h. The lysates were incubated with antibodies overnight at 4 °C, and then with 25 μl protein G-Sepharose beads for 2 h at 4 °C. The beads were washed with the lysis buffer, and proteins eluted in sample buffer. Flag-tagged RelB was immunoprecipitated with anti-Flag-M2 beads and eluted with Flag peptide (Sigma).

### Generation of SIRT1 overexpressing cells

To generate stable clones, 4 μg SIRT1 expression plasmid were transfected into U373 cells. Clones selected in DMEM containing 0.4 μg/ml G418 were subsequently pooled.

### Glycolysis assay

Glycolysis in cultured cells was measured exactly as described before^54^. The conversion of D-[5-^3^H(N)]-glucose to ^3^H_2_O was calculated, and expressed as glycolytic rate (% glucose conversion/10^6^ cells/6 h).

### Unbiased cytokine Kaplan–Meier analysis

Cytokine and Cytokine Receptor *Z*-score normalized expression data was downloaded from TCGA^55^. Patients were divided into high and low expressing groups based on the mean value of gene expression. Kaplan–Meier analysis was performed for each gene based on high and low expression groups. Cytokines/receptors were ranked based on the statistical significance of the individual Kaplan–Meier analysis. Rank of cytokines and cytokine receptors were combined to create a combined rank score to indicate the prognostic impact of each cytokine signaling program.

### Fractionation

Cells were washed with cold PBS and re-suspended in buffer containing 10 mM Hepes (pH 7.8), 10 mM KCl, 0.1 mM EDTA, 1 mM sodium orthovanadate, 1 mM DTT, 1:500 protease inhibitors (Sigma), and 0.2 mM PMSF, and incubated on ice for 15 min. NP-40 was added (to 0.75%) and cells were vortexed for 10 s. Nuclei and cytoplasm were separated by centrifugation at 3000 rpm for 3 min at 4 °C. Nuclei were re-suspended in buffer containing 20 mM Hepes (pH 7.8), 0.4 M NaCl, 1 mM EDTA, 1 mM sodium orthovanadate, 1 mM DTT, and protease inhibitor coctail and incubated on ice for 15 min. Nuclear extracts were cleared by centrifugation at 14,000×*g* for 5 min at 4 °C.

### Generation of U373-RelB^−/−^ cells

Guide RNAs (5′-caccgGGTCTGGCGACGCGGCGACT-3′ and 5′-aaacAGTCGCCGCGTCGCCAGACCc-3′) targeting RelB gene were designed using the MIT CRISPR guide design tool (http://crispr.mit.edu/). Guide RNA was cloned into BsmBI-digested LentiCRISPRV2 (Addgene). Viral particles were packaged in HEK293T cells using standard approaches. U373 cells were infected, selected in medium containing 75 μg/ml puromycin, and individual colonies were isolated and screened for homozygous RelB knockout. The absence of the off-targets was verified by PCR amplification of predicted off-target fragments and sequencing.

### Migration

Cells were cultured in six-well plates and serum starved overnight. Cells were stimulated, wounded, and pictures were taken at time 0 and 24 h. Cells migrating past original wound boundary were enumerated and reported.

### Proliferation

Proliferation was examined using WST-8 Cell Proliferation Assay (Dojindo Molecular Technologies).

### SIRT1 activity assay

SIRT1 activities were determined using fluorometric kit (Abcam). Cells were lysed in lysis buffer and then sonicated on ice. 200 μg of cell lysate was incubated with anti-SIRT1 antibodies overnight at 4 °C and then with 20 μl of 50% Protein A beads for 3 h at 4 °C. Beads were washed in SIRT1 assay buffer (50 mM Tris pH 8.8, 0.5 mM DTT). Activity of immunoprecipitated SIRT1 was determined using flouro–substrate mixture according to instructions, and absorbance at 455 nm was tabulated.

### Clinical samples

Both patient tissue and RNA samples were provided by the VCU Tissue Acquisition and Analysis core.

### Immunoflouresence

Cells cultured on coverslips were washed with PBS and fixed in 2.5% paraformaldehyde for 10 min at room temperature. Cells were washed with 0.3 M glycine and permeabilized with 0.1% Triton X-100. Coverslips were blocked with 5% BSA/1% normal goat serum for 1 h, incubated with primary antibodies overnight at 4 °C, washed with PBS and incubated with secondary antibodies for 1 h. Coverslips were washed again, counterstained with Hoescht and mounted using VectaShield (Vector Laboratories).

### Immunohistochemistry

Slides were fixed in ice cold acetone for 20 min, incubated in 1% hydrogen peroxide, washed in PBS, and blocked in 5% BSA/1% normal goat serum for 1 h. The slides were then incubated with primary antibodies in blocking buffer overnight at 4 °C. Slides were subsequently washed in PBS and incubated with EnVision^+^ secondary reagent (Agilent) for 20 min, washed in PBS, and then exposed using DAB^+^ chromogen (Agilent) for 10 min.

### Microarray processing and differential expression analysis

Microarrays were processed using the R statistical package requiring the libraries ‘affy’, ‘affyPLM’. Quality control was performed using analysis of 3′/5′ ratios, generating representative array images, and creating RNA degradation plots after generating affybatch objects using the ‘affy’ and ‘affPLM’ libraries. Expression summaries of arrays were generated after background correction, and normalization using robust multichip averaging. Statistical significance was assessed using S-testing.

### RNA-seq processing and differential expression analysis

RNA was isolated using the ‘mirVana RNA isolation kit’ (ThermoFisher Scientific). RNA was sent to the University of Cincinnati Genomics Core for quality control and RNA-sequencing analysis. Data was analyzed using the tuxedo pathway, fastq read files were aligned using BowTie2, transcript assembly, and FPKM estimates achieved using CuffLinks, and testing for differential gene expression and promoter usage were accomplished using CuffDff.

### ChIP assay

The cells were cross-linked with 1% formaldehyde for 10 min at 37 °C and washed with ice-cold PBS containing 125 mM glycine. Chromatin was sheared using a Diagenode Bioruptor (Liège) on high setting for two 10 min intervals (30 s on/off). Anti-SIRT1, anti-YY1 (both Santa Cruz Biotechnology), and anti-acH3 (Millipore) antibodies were used. DNA was detected by qPCR using TaqMan primers: IL1B forward: 5′-aatttaaaacattcttctaacgtggg-3′, reverse: 5′-ggagtagcaaactatgacacattttg-3′, and probe: 5′-[6-FAM] caactgcacaacgattgtcaggaaaa[BHQ1a-Q]-3′; IL8 forward: 5′-gtgcataagttctctagtagggtgatg-3′, reverse: 5′-ggctcttgtcctagaagcttgtgt-3′, and probe: 5′-[6-FAM]cactccataaggcacaaactttcagag[BHQ1a-Q]-3′; Chi3L1 forward: 5′-gtgcagccgccccgta-3′, reverse: 5′-gcaatttacatgctgattatttagaggg-3′, and probe: 5′-[6-FAM]gcaaaatagcaccggggcttaaag[BHQ1a-Q]-3′. qPCR data was calculated as percent input; IgG was used as a control.

### Pearson correlation and gene copy number analysis

Genome-wide analysis of Pearson correlation (with IL-1β expression) and Identification of Copy Number Variation was conducted using the co-expression functionality and GISTIC, respectively, for the data downloaded from the CBioPortal to TCGA^55^.

### Patient stratification, differential gene expression, pathway enrichment, and prognostic significance analyses

*Z*-score normalized RelB expression data was downloaded from TCGA via cBioPortal (*n* = 206). Patients with *Z*-score of >0.25 or <−0.25 were classified as high and low expressors. Differential gene expression testing was conducted using the ‘limma’ library of the R statistical package. Genes upregulated in patients expressing high RelB levels were used for pathway enrichment analysis using the EnrichR software package^37^. Prognostic significance was established via Kaplan–Meier analysis, comparing patients expressing RelB above of or below the mean level of gene expression.

### Promoter analysis and identification of YY1

2.5 kb-long regions surrounding the transcription start sites of the RelB-controlled genes were downloaded from the hg37 genome using the UCSC browser, and analyzed for the presence of regulatory elements (JASPAR and TRANSFAC databases) using the ‘PathView’ and ‘EnrichR’ R-packages (RelB-controlled vs. a random genes).

### CytoAnalysis

CIBERSORT analysis was conducted using default settings as described^56^.

### Statistical analysis

All experiments were repeated at least three times with consistent results. Data are presented as mean ± SD. Statistical analysis was performed using GraphPad Prism 7. Values are displayed as mean ± standard deviation. *T*-tests and ANOVAs were performed as indicated. Sidak’s or Tukey’s test was performed to compare multiple groups.

## Supplementary information

supplemental table 1

supplemental figure 1

supplemental figure 2

supplemental figure 3

supplemental figure 4

Supplementary FIGURE LEGENDS
