# Supplementary material for: RelB acts as a molecular switch driving chronic inflammation in glioblastoma multiforme
Source: Oncogenesis. 2019 May 29;8(6):37. doi: 10.1038/s41389-019-0146-y (PMC6541631; doi:10.1038/s41389-019-0146-y)
Supplement: Supplementary file 1 — supplemental table 1 [file 41389_2019_146_MOESM1_ESM.pdf]

Supplementary table I

Cytokine	Receptor Gene	Cytokines	Receptors	Combined	Enrichment Score
**IL1B**	**IL1R1**	0.002	0.007	0.000	**3.775**
**OSM**	**OSMR**	0.001	0.012	0.000	**3.751**
**TNFSF11**	**TNFRSF11A**	0.051	0.002	0.001	**3.052**
**IL6**	**IL6R**	0.013	0.006	0.001	**3.169**
**TNF**	**TNFRSF1A**	0.086	0.001	0.001	**2.965**
**IL4**	**IL4R**	0.062	0.005	0.002	**2.618**
**IL1A**	**IL1R1**	0.047	0.007	0.002	**2.646**
**IL18**	**IL18R1**	0.099	0.004	0.003	**2.539**
**TNFSF13B**	**TNFRSF17**	0.000	0.093	0.000	**3.736**
**TNFSF13**	**TNFRSF17**	0.001	0.093	0.001	**3.292**
**IL10**	**IL10RA**	0.034	0.029	0.006	**2.215**
**CSF1**	**CSF1R**	0.207	0.002	0.004	**2.454**
**IL15**	**IL15RA**	0.166	0.004	0.005	**2.310**
**CSF3**	**CSF3R**	0.085	0.017	0.008	**2.076**
**IL7**	**IL7R**	0.062	0.036	0.012	**1.921**
**TNFSF12**	**TNFRSF12A**	0.108	0.013	0.009	**2.065**
**CD70**	**CD27**	0.067	0.047	0.016	**1.794**
**IL20**	**IL20RA**	0.115	0.017	0.011	**1.954**
**TNFSF14**	**LTBR**	0.007	0.311	0.014	**1.846**
**TGFB2**	**TGFBR2**	0.515	0.003	0.013	**1.880**
**KITLG**	**KIT**	0.033	0.300	0.049	**1.313**
**LTA**	**TNFRSF1A**	0.948	0.001	0.074	**1.132**
**TNFSF10**	**TNFRSF10A**	0.025	0.330	0.043	**1.363**
**FASLG**	**FAS**	0.265	0.022	0.031	**1.513**
**LIF**	**LIFR**	0.011	0.482	0.038	**1.423**
**TGFB3**	**TGFBR3**	0.013	0.788	0.113	**0.946**
**IL2**	**ISG20**	0.465	0.033	0.080	**1.095**
**IL13**	**IL13RA1**	0.417	0.040	0.081	**1.091**
**IL17A**	**CDw217**	0.173	0.107	0.071	**1.147**
**TGFB1**	**TGFBR1**	0.309	0.076	0.093	**1.032**
**MST1**	**MST1R**	0.110	0.311	0.122	**0.913**
**IL11**	**IL11RA**	0.102	0.365	0.135	**0.871**
**TNFSF8**	**TNFRSF8**	0.447	0.112	0.177	**0.753**
**TNFSF18**	**TNFRSF18**	0.755	0.057	0.245	**0.611**
**LTB**	**LTBR**	0.247	0.311	0.218	**0.662**
**EPO**	**EPOR**	0.598	0.160	0.302	**0.520**
**TPO**	**MPL**	0.586	0.218	0.350	**0.455**
**IFNG**	**IFNGR1**	0.603	0.196	0.341	**0.467**
**IFNA1**	**CD118**	0.207	0.482	0.280	**0.553**
**IL5**	**CDw125**	0.706	0.360	0.550	**0.260**
**CSF2**	**IL5RA**	0.808	0.360	0.639	**0.195**
**IFNB1**	**CD118**	0.736	0.482	0.653	**0.185**
**FLT3LG**	**FLT3**	0.955	0.426	0.867	**0.062**
