# Supplementary figures and images for: RelB acts as a molecular switch driving chronic inflammation in glioblastoma multiforme
Source: Oncogenesis. 2019 May 29;8(6):37. doi: 10.1038/s41389-019-0146-y (PMC6541631; doi:10.1038/s41389-019-0146-y)

Supplementary Fig. 1

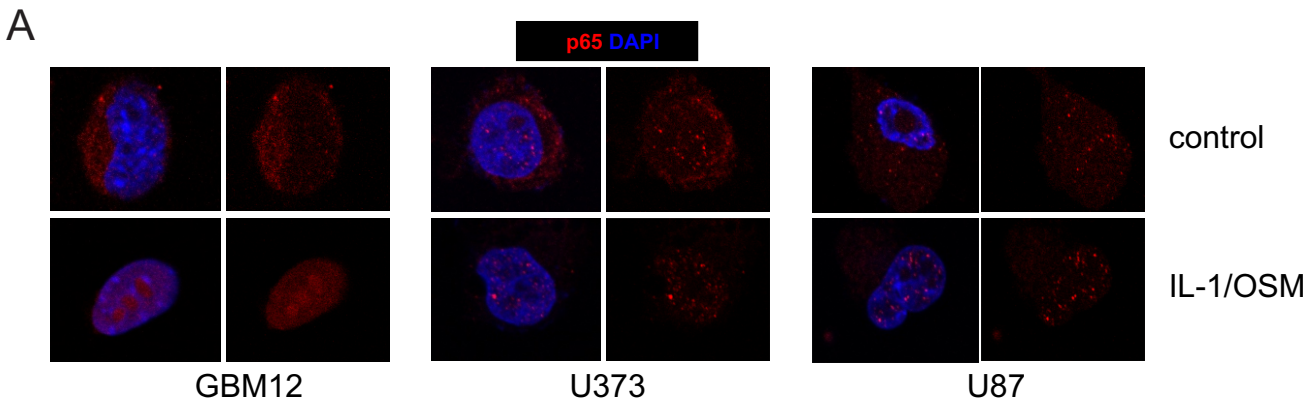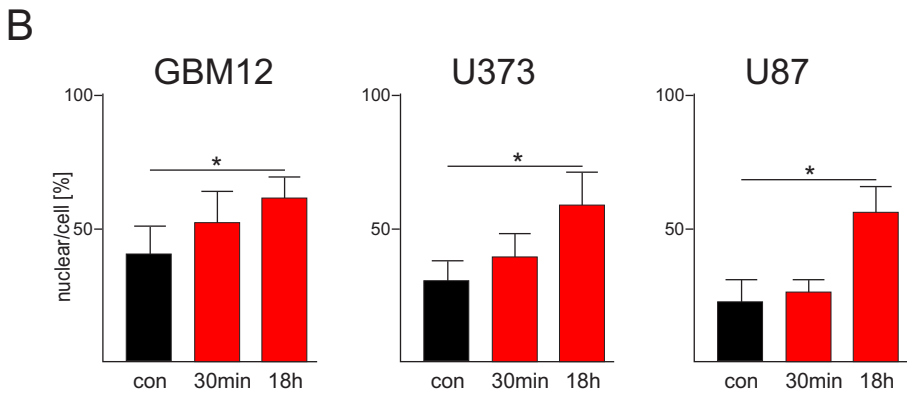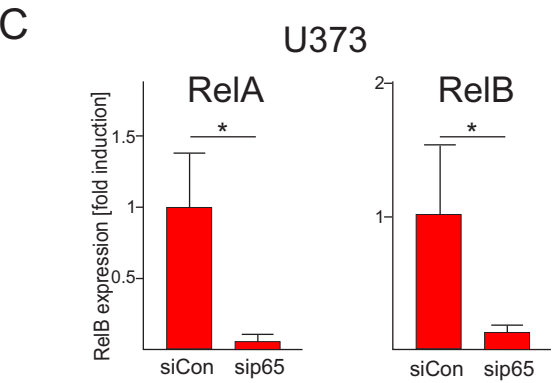

Supplement: Supplementary file 2 — supplemental figure 1 [file 41389_2019_146_MOESM2_ESM.pdf]

Supplementary Fig. 2

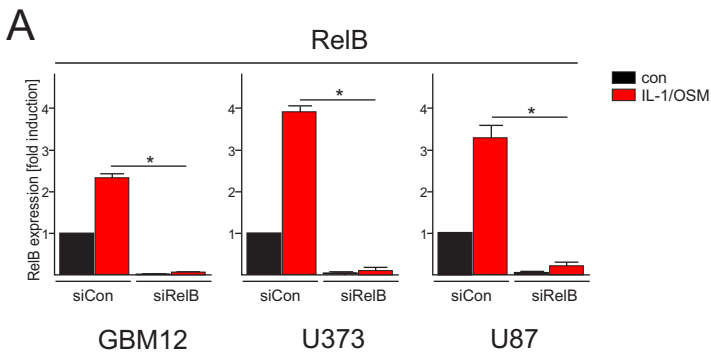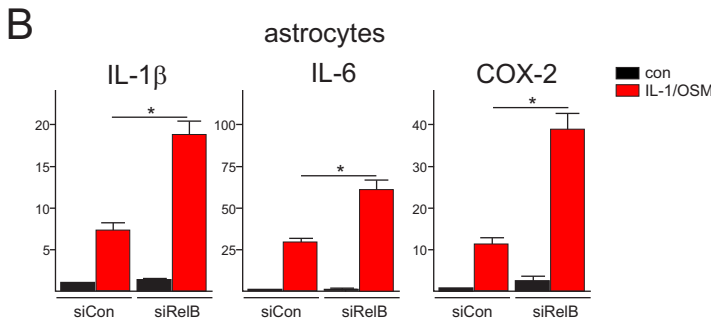

Supplement: Supplementary file 3 — supplemental figure 2 [file 41389_2019_146_MOESM3_ESM.pdf]

Supplementary Fig. 3

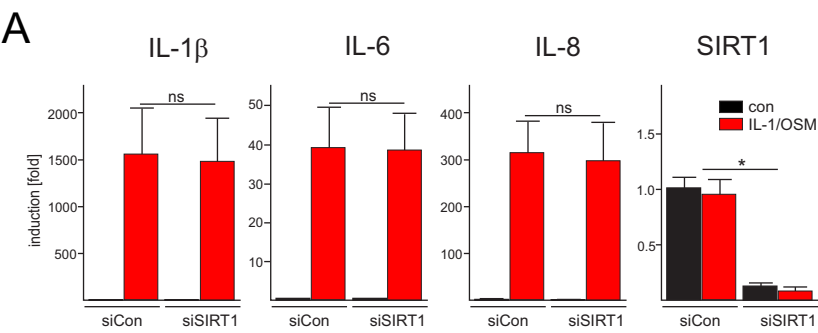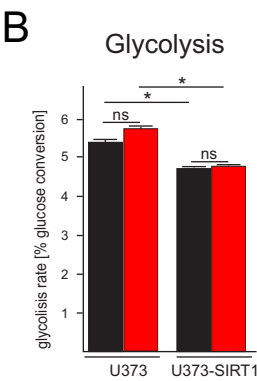

Supplement: Supplementary file 4 — supplemental figure 3 [file 41389_2019_146_MOESM4_ESM.pdf]

Supplementary Fig. 4

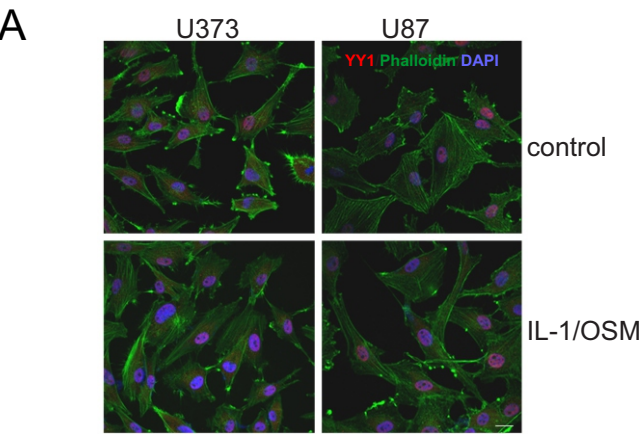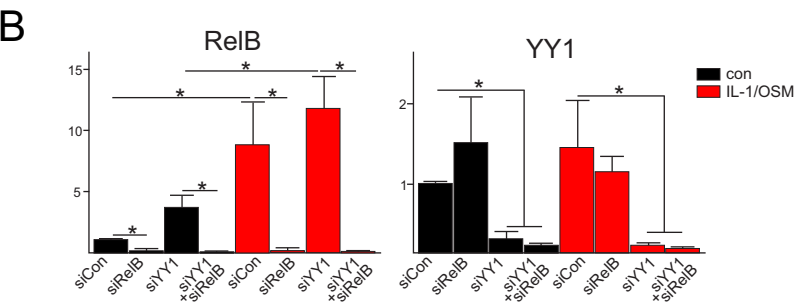

Supplement: Supplementary file 5 — supplemental figure 4 [file 41389_2019_146_MOESM5_ESM.pdf]
