# Supplementary material for: RelB acts as a molecular switch driving chronic inflammation in glioblastoma multiforme
Source: Oncogenesis. 2019 May 29;8(6):37. doi: 10.1038/s41389-019-0146-y (PMC6541631; doi:10.1038/s41389-019-0146-y)
Supplement: Supplementary file 6 — Supplementary FIGURE LEGENDS [file 41389_2019_146_MOESM6_ESM.pdf]

SUPPLEMENTARY FIGURE LEGENDS

Supplementary Table 1. Inflammatory signature of GBM patients. Patient expression data were obtained from TCGA (n=208). The mean Z-score normalized expression values for cytokine and cytokine receptor genes were used to group patients into high and low expressors. Kaplan Meier analysis was performed yielding a p-value for each gene and survival. These p-values were used to rank cytokines and cytokine receptors as indicators of poor prognosis. A combined prognostic score was then generated for each cytokine and its receptor.

Supplementary Figure 1. p65 and RelB in GBM cells. (A) GBM cells were stimulated with IL-1/OSM for 18h and localization of p65 was analyzed by immunofluorescence. DAPI was used to visualize nuclei. (B) Images shown if Fig. 2D were imported into ImageJ and RelB foci were quantified. “Find Maxima” tool was used with a prominence of >5-30. n = 4-10 cells in 5 fields. (C) U373 cells were transfected with the indicated siRNAs, stimulated 48h later with IL-1/OSM for 18h. Expression was analyzed by qPCR (three experiments, error bars represent s.d., * $p<0.05$ (T-test, Sidak’s test).

Supplementary Figure 2. Knock-down of RelB in GBM cells and astrocytes. GBM cells (A) or primary human astrocytes (B) were transfected with the indicated siRNAs, stimulated 48h later with IL-1/OSM for 18h. Expression was analyzed by qPCR (three experiments, error bars represent s.d., * $p<0.05$ (two-way ANOVA, Sidak’s test).

Supplementary Figure 3. SIRT1 in GBM cells. (A) U373 cells transfected with the indicated siRNAs were stimulated 48h later with IL-1/OSM for 18h. Expression was analyzed by qPCR

(three experiments, error bars represent s.d., * $p<0.05$ (two-way ANOVA, Sidak's test). (B) U373 cells were stimulated with IL-1/OSM for 8h, labelled with D-[5-^{3}H(N)]-glucose for 5 hours, and glycolysis rate was calculated as % glucose conversion/5h/10^6 cells.

Supplementary Figure 4. RelB and YY1 in GBM cells. (A) U373 and U87 cells were stimulated with IL-1/OSM for 18h, and analyzed by immunofluorescence, using YY1 antibodies. Cells were also stain with Phalloidin and DAPI. (B) U373 cells transfected with the indicated siRNAs were stimulated 48h later with IL-1/OSM for 18h. Expression was analyzed by qPCR (three experiments, error bars represent s.d., * $p<0.05$ (two-way ANOVA, Sidak's test).
